# Synergistic Effects and Sex Differences in Anthropometric Measures of Obesity and Elevated High-Sensitivity C-Reactive Protein Levels

**DOI:** 10.3390/ijerph17218279

**Published:** 2020-11-09

**Authors:** Fatima Nari, Bich Na Jang, Gyu Ri Kim, Eun-Cheol Park, Sung-In Jang

**Affiliations:** 1 Department of Public Health, Graduate School, Yonsei University, Seoul 03722, Korea; fatima@yuhs.ac (F.N.); jbn2846@yuhs.ac (B.N.J.); 2 Institute of Health Services Research, Yonsei University, Seoul 03722, Korea; ecpark@yuhs.ac; 3 Department of Preventive Medicine, Yonsei University College of Medicine, Seoul 03722, Korea; GYURIKIM@yuhs.ac

**Keywords:** BMI, waist circumference, interaction analysis, inflammation, cardiovascular risk

## Abstract

*Background:* It remains unclear which anthropometric measure best predicts elevated high-sensitivity C-reactive protein (hs-CRP) levels. This study investigated the association and synergistic interaction of two obesity indices with elevated hs-CRP levels in a national sample of Korean adults, stratified by sex. *Methods:* The present cross-sectional study used data from the 2015–2018 Korea National Health and Nutrition Examination Survey of 18,610 subjects aged ≥20 years after excluding those with missing variables. Multiple logistic regression analyses and chi-squared tests were performed to investigate the association between body mass index (BMI) and waist circumference (WC) with elevated hs-CRP levels. Interaction analysis was used to examine the synergistic effect between BMI and WC on the risk of having elevated hs-CRP levels. *Results:* Elevated hs-CRP levels exceeding 3 mg/L were present in 9.3% and 7.5% of men and women, respectively. The relationship between each obesity index and elevated hs-CRP levels was significant in women (high WC (odds ratio [OR] = 1.77, 95% confidence interval [CI] = 1.24–2.54), high BMI (OR = 2.08, 95% CI = 1.58–2.74)) but not in men (high WC (OR = 1.19, 95% CI = 0.86–1.64), high BMI (OR = 0.99, 95% CI = 0.77–1.29)). Furthermore, combined measures of the two obesity indices and interaction analysis results revealed a synergistic association in men (OR = 1.57, 95% CI = 1.33–1.85; relative excess risk due to interaction (RERI) = 0.39, 95% CI = −0.09–0.86), and women (OR = 3.70, 95% CI = 3.09–4.43; RERI = 0.85, 95% CI = −0.06–1.75). *Conclusion:* BMI and WC were significantly associated with a risk of elevated hs-CRP levels in women but not in men. Nevertheless, significant synergistic interactions were seen in combined measures of BMI and WC, regardless of sex. These findings emphasize the need to use both measures of adiposity concurrently in the assessment of obesity and when identifying cardiovascular risk.

## 1. Introduction

Obesity is a chronic disease that poses a great global burden, as it affects over one billion adults throughout the world [1]. In South Korea, the prevalence of obesity in the population was estimated to be 32.4% in 2015 [2,3].

Obesity has been linked to an increased risk of many different illnesses, including but not limited to hypertension, metabolic syndrome, coronary heart disease, and other atherosclerotic diseases [4]. Additionally, elevated high-sensitivity C-reactive protein (hs-CRP), as an indicator of low-grade inflammation, has been considered a possible risk factor for cardiovascular diseases [5] associated with obesity and visceral adiposity [6]. 

Hs-CRP levels have emerged as a useful biomarker for atherosclerotic processes and future cardiovascular disease (CVD) risk [7], acting as a more sensitive alternative to the traditional CRP marker [8,9,10]. Evidence suggests that adipose tissue is a major source of inflammatory cytokines, such as tumor necrosis factor-α and interleukin-6. In turn, the presence of these cytokines in the circulation stimulates inflammation and increases CRP levels in the liver [11]. Thus, a strong positive association has been found between measures of obesity, such as waist circumference (WC) and body mass index (BMI), and CRP levels [12,13]. 

However, with respect to the association between hs-CRP levels and obesity, prior studies have shown that the relationship differs according to various factors, such as age, sex, and ethnicity [14]. Additionally, normal-weight individuals with metabolic dysfunction have greater cardiovascular risk than metabolically healthy obese subjects in the Asian population when assessed using BMI [15]. This indicates that one measure of obesity, such as BMI alone, cannot precisely quantify health risks in different subgroups and populations [14].

Consequently, prior studies have advocated for the use of multiple measures of obesity rather than one independent measure of obesity in assessing adverse health outcomes, such as cardiovascular risk. A study carried out in the Norwegian adult population showed that the combined measure of BMI and WC resulted in a worse prognosis of health indicators and lifestyle factors, such as unhealthy diet, low physical activity, and elevated cardiovascular risk [16]. Ki et al. deemed that the use of BMI and WC are both equally effective measures in identifying multiple cardiovascular risk factors in the Korean population [17]. The study concluded that each individual index of obesity showed a significant, albeit modest, association with cardiovascular risk factors. The authors then went on to recommend the use of both indices to provide more insight regarding cardiovascular risk in Korean men and women [17].

Thus, our present study’s primary objective was to investigate the association between two obesity indices, BMI and WC, and their combination with elevated hs-CRP levels in Korean adults. Our secondary aim was to highlight any synergistic interactions of the use of combined BMI-WC measures in our study population.

## 2. Materials and Methods 

Our study used data derived from the 2015 to 2018 Korean National Health and Nutrition Examination Survey (KNHANES). The KNHANES is a cross-sectional survey that uses a complex multistage sampling method to analyze approximately 10,000 people annually. We excluded those younger than 20 years because we deemed that the recommended guidelines for obesity assessment in pediatric individuals [18] and adults above 20 years are very different. Furthermore, after excluding those with missing data, we included 18,610 people in our study.

The main outcome measure in our study was elevated hs-CRP levels. According to the criteria set by the American Heart Association and Centers for Disease Control and Prevention, an hs-CRP level of >3.0 mg/L is defined as reflecting a high risk of developing future cardiovascular disease [19].

To classify general obesity, we used the criteria stipulated by the World Health Organization (WHO) for general obesity in the Asian population [20]: A BMI higher than 25 kg/m^2^. To classify central obesity, we used high WC as defined by the Korean Society for the Study of Obesity (KSSO). The recommended cut-off point for waist circumference was ≥90 cm for women and ≥85 cm for men [21].

Data regarding sociodemographic characteristics and health behavior-related variables were all added as potential confounders in this study. Sociodemographic characteristics included sex, age (20–34 years, 35–49 years, ≥50 years), education level (middle school degree or lower, high school degree, university degree or higher), and income level per month, which was divided into four categories (low, middle low, middle high, and high). Additionally, marital status was divided into married and unmarried, and region was divided into urban and rural. Job classification was based on the Korean Standard Occupational Classification (sixth revision) and was divided into four categories: White collar workers (office work), pink collar workers (sales and service), blue collar workers (agriculture, forestry, fishery, and armed forces occupation) [22], and unemployed.

Smoking experience and alcohol drinking experience were classified as Never and Ever. Aerobic exercise was classified based on the validated Korean version of the International Physical Activity Questionnaire (IPAQ) [23]. Those who were coded in the “Yes” group were defined as those who met recommended levels of aerobic exercise, which was up to 150 min per week of moderate-intensity exercise or up to 75 min per week of vigorous exercise. Otherwise, they were coded into the “No” group. Chronic diseases included in this study were hypertension, diabetes mellitus, stroke, and myocardial infarction, and the number of comorbidities were grouped into three categories: 0 comorbidities, 1 comorbidity, and ≥2 comorbidities. Subjective health condition was divided into Good, Normal, and Bad.

Multivariable logistic regression was used to analyze the association between general and central obesity with elevated hs-CRP levels in the study population, which were also carried out in subgroups. *P*-values less than 0.05 were considered statistically significant. The three additive interaction measures used in our study were relative excess risk due to interaction (RERI); the proportion of disease among those with both exposures that is attributable to their interaction, i.e., Attributable Proportion (AP); and Synergy Index (SI). We also estimated their corresponding 95% confidence interval (CI) using the method described by Homer and Lemeshow [24]. They were calculated according to the following equations: RERI = OR11 − OR10 − OR01 + 1, AP = RERI/OR11, and SI = (OR11–1)/(OR01–1) + (OR10–1) [25]. If RERI and AP did not equal 0 and SI exceeded unity, then a synergistic effect was considered present. In contrast, if RERI was less than 0, then the interaction effect between the two exposures was considered to be antagonistic [25]. All statistical analyses were conducted using SAS 9.4 software (SAS Inc., Cary, NC, USA).

## 3. Results

The study population’s general characteristics are shown in Table 1. In total, 18,610 people were included in this study, of which 8842 were men and 9768 were women. The prevalence of elevated hs-CRP in those with high WC was 11.0% for men and 8.0% for women, and in those with high BMI, it was 7.2% and 9.0% in men and women, respectively. Men and women with both high BMI and high WC had a prevalence of elevated hs-CRP of 12.0% and 15.3%, respectively. 

Table 2 shows the association of BMI and WC with the risk of elevated hs-CRP levels. Women with high WC or high BMI had a statistically significant higher risk of elevated hs-CRP levels than those in the normal range, while men did not exhibit any significant association (men: High WC (odds ratio [OR] 1.19, 95% CI 0.86–1.64), high BMI (OR 0.99, 95% CI 0.77–1.29); women: WC (OR 1.77, 95% CI 1.24–2.54), high BMI (OR 2.08, 95% CI 1.58–2.74)). Furthermore, participants who had concurrent high BMI and high WC had the greatest risk of elevated hs-CRP levels compared to those in the normal range (men: OR 1.57, 95% CI 1.33–1.85; women: OR 3.70, 95% CI 3.09–4.43). The additive interaction results in men were as follows: RERI was 0.39 (95% CI, −0.09–0.86), SI was 3.16 (95% CI 2.69–3.64), and AP was 0.25 (95% CI −0.23–0.72). The results in women were as follows: RERI was 0.85 (95% CI −0.06–1.75), SI was 1.46 (95% CI 0.55–2.36), and AP was 0.23 (95% CI −0.68–1.13).

**Figure 1:**
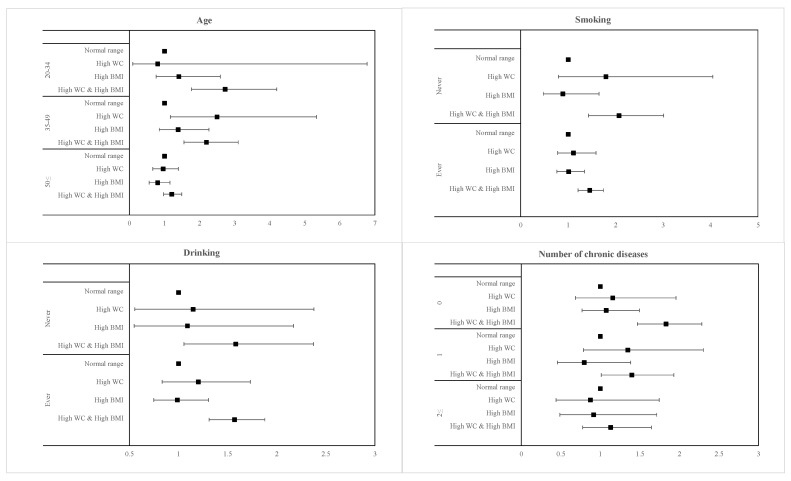
Forest plots of subgroup analysis results in men.

**Figure 2:**
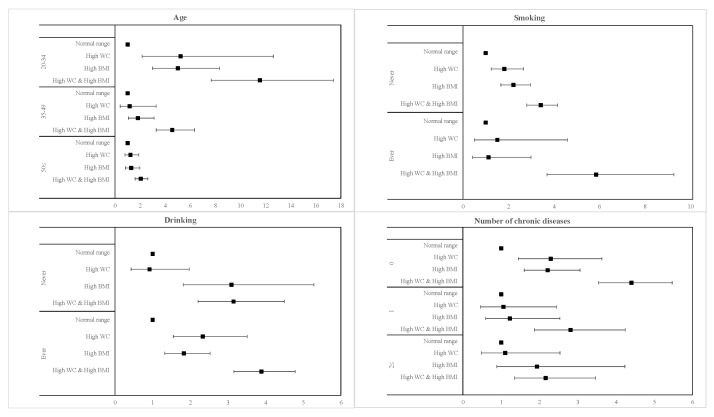
Forest plots of subgroup analysis results in women.

The subgroup analysis of the relationship of elevated hs-CRP risk with age, smoking, drinking, and number of chronic diseases is presented in Figure 1 and Figure 2 for men and women, respectively, with high BMI and high WC. Generally, men and women who had both high BMI and high WC were more significantly associated with all variables than either measure of adiposity individually. In addition, men with both high BMI and high WC and who were younger (20–34 years; OR 2.73, 95% CI 1.77–4.20), never smokers (OR 2.07, 95% CI 1.43–3.01), and who were seemingly healthy with no presence of comorbidities (OR, 1.83, 95% CI 1.47–2.28) had a higher risk of elevated hs-CRP levels than their respective counterparts. There was no noticeable difference in the risk of elevated hs-CRP between never and ever drinkers in men. Similarly, for women with concurrent high BMI and high WC and who were younger (20–34 years; OR 11.55, 95% CI 7.67–17.40), ever smokers (OR 5.86, 95% CI 3.70–9.27), ever drinkers (OR 3.89, 95% CI 3.16–4.79), and who were seemingly healthy with no presence of comorbidities (OR 4.40, 95% CI 3.55–5.47) had a higher risk of elevated hs-CRP levels than their respective counterparts.

## 4. Discussion

Our present study utilized cross-sectional data from KNHANES to investigate the association of obesity and central obesity with elevated hs-CRP levels. Our findings suggest that while obesity and central obesity were both individually associated with elevated hs-CRP levels in women, such a relationship was not present in men. Moreover, those with both obesity indices had the highest risk of elevated hs-CRP levels in Korean men and women compared to either individual index of obesity. Our findings confirm previous research findings concerning the relationship between the adiposity measures of WC and BMI and systemic inflammation [12,26]. 

Perhaps one of the most significant findings of our present study was the sex difference between measures of adiposity and hs-CRP levels. Female individuals consistently exhibited a higher risk of elevated hs-CRP levels for both measures of obesity than men, both individually and combined. Our findings are supported by a number of other studies, where a strong correlation for both WC and BMI with hs-CRP was seen in women [13,26]. In contrast to our study’s results, an investigation carried out in the Korean population regarding the effects of individual BMI and WC indices on cardiovascular risk identified significant relationships in both sexes [17]. Nevertheless, a prior study in Korea showed that sarcopenic obesity was linked to elevated hs-CRP levels in women but not in men [27]. In a multivariate analysis study carried out in Mediterranean women with obesity, BMI and WC were independently correlated with hs-CRP concentration [28]. Whereas there is no concrete evidence regarding the sex disparity in the relationship between obesity and elevated hs-CRP levels, two mechanisms have been suggested. First, the secretion of estrogen in female individuals may play a role in the etiology of inflammation [29], thereby leading to a more pronounced effect on hs-CRP levels. Second, women generally have higher levels of body and visceral fat than men [30], with a consequent elevation in hs-CRP levels and cardiovascular risk.

Whereas the use of BMI as an obesity index has proven valuable and carries great implication [31], it also comes with its share of limitations. As previously mentioned, at a similar BMI, women have a greater percentage of body fat than men. The use of another measure of obesity, namely WC, to increase the reliability of adiposity evaluation, is encouraged. Our study’s data reflect the difference in the association of obesity indices, showing a higher prevalence of elevated hs-CRP levels in women than in men among people with obesity. Thus, in accordance with prior findings, the presence of a high BMI and abdominal obesity as measured using WC and their influence on the risk of elevated hs-CRP levels was significant in both men and women [16].

To the best of our knowledge, this study is one of the few that carried out an interaction analysis to examine the possible additive interaction between high BMI and high WC with respect to elevated hs-CRP levels in the Korean population. The results of our study revealed that the effect of either factor on elevated hs-CRP levels was significantly increased by the other, which is consistent with an additive interaction [32]. The European guidelines for the management of obesity also concluded that greater BMI is associated with higher metabolic and CVD risk due to the accumulation of intra-abdominal fat, as measured by waist circumference [33]. Likewise, the 2018 guidelines for obesity published by the KSSO revealed that the risk of comorbidities, such as diabetes and hypertension, was severe in individuals with a BMI ≥25 kg/m^2^ and with abdominal obesity [34]. According to the WHO, the rationale for a lower BMI cut-off point for obesity (≥25 kg/m^2^) is that Asians generally have a greater percentage of body fat compared to non-Asians at the same BMI [20,34].

In the subgroup analysis, men and women in their 20s with concurrent general and central obesity had the highest risk of elevated hs-CRP levels, and the risk decreased with increasing age. This finding is consistent with those of several other studies. One study stated that older women with obesity were less likely to have clinically raised hs-CRP levels than younger women with obesity, although this trend was not seen in men [31]. Park et al. showed that obesity in younger subjects, especially those younger than 60 years of age, were more likely to have elevated hs-CRP levels than older subjects [27]. A study carried out in younger Koreans revealed that obese individuals were positively associated with hs-CRP levels in the upper tertile (i.e., >0.5 mg/L) [35]. In addition, while obesity in younger and middle-aged adults is associated with higher cardiovascular risk and other negative health outcomes, this does not seem to be the case in older adults [27,36]. In fact, high BMI in the aging population carries a somewhat protective effect, and while the mechanism is not fully understood, it is associated with a decreased risk of morbidity and death [37]. 

Furthermore, while smoking and drinking did not show a statistically significant association in men, it was associated with elevated hs-CRP levels in women. In contrast, the relationship between smoking, obesity, and markers of inflammation has already been established in prior studies. A study by Wafika et al. suggested that hs-CRP levels in men who were smokers and were both obese and had central obesity were higher than those in smokers with normal anthropometric values [38]. Additionally, another study by Mazen et al. carried out in women revealed that there were no significant differences in hs-CRP levels between smokers and nonsmokers [39,40].

Another point to note from our subgroup analysis results is the fact that individuals who were seemingly healthy were at a higher risk of having elevated hs-CRP levels than those who had one or more chronic diseases. This finding could be supported by a study that revealed that hs-CRP levels were higher in those who had diabetes than those who did not. An explanation that was offered was that diabetics take medication, which could have lowered the level of hs-CRP in those individuals [41]. Therefore, the finding that healthier individuals had a higher risk of elevated hs-CRP levels may be attributed to the fact that those who were sick took medications and were more health-conscious.

This study has several limitations. First, as this was a cross-sectional study, we could not infer the causality and temporality of any relationship. Specifically, the directionality between the two obesity indices and inflammation could not be determined. In other words, we cannot be sure whether elevated hs-CRP levels were a cause or consequence of obesity. Second, we used a single assessment of cardiovascular risk, hs-CRP levels, in our study, which may have affected the variability of our study.

Nevertheless, our study’s main strength lies in the fact that we investigated the synergistic relationship and sex differences between the combined obesity indices of BMI and WC and elevated hs-CRP levels. Second, our study included a large and homogenous national sample and investigated the sex differences in the association of BMI and WC with elevated hs-CRP levels.

## 5. Conclusions

Our study’s findings have important methodological implications, with conclusions that both BMI and WC were independently associated with elevated hs-CRP levels in women but not in men. In addition, our study provides novel information, as a synergistic interaction effect was seen with concurrent BMI and WC and presented a higher risk of elevated hs-CRP levels in both sexes.

Hence, considering the public health burden of both obesity and elevated hs-CRP levels, obesity-targeted prevention and management strategies are warranted to prevent and ameliorate future inflammation and risk of CVD. 

## Figures and Tables

**Table 1:** General characteristics of study population (total = 18,610 people).

Variables	Total		Male					Total		Female				
			Elevated hs-CRP Levels ≥3 mg/L				*p*-Value			Elevated hs-CRP Levels ≥3 mg/L				*p*-Value
			Yes		No					Yes		No		
	*n*	(%)	*n*	(%)	*n*	(%)		*n*	(%)	*n*	(%)	*n*	(%)	
Total	8842	(100.0)	818	(9.3)	8024	(90.7)		9768	(100.0)	732	(7.5)	9036	(92.5)	
Obesity							<0.0001							<0.0001
Normal	4786	(54.1)	387	(8.1)	4399	(91.9)		6409	(65.6)	302	(4.7)	6107	(95.3)	
High WC ^a^	427	(4.8)	47	(11.0)	380	(89.0)		476	(4.9)	38	(8.0)	438	(92.0)	
High BMI ^b^	1069	(12.1)	77	(7.2)	992	(92.8)		781	(8.0)	70	(9.0)	711	(91.0)	
High BMI & WC	2560	(29.0)	307	(12.0)	2253	(88.0)		2102	(21.5)	322	(15.3)	1780	(84.7)	
Age							<0.0001							0.6737
20–34	1766	(20.0)	118	(6.7)	1648	(93.3)		1995	(20.4)	160	(8.0)	1835	(92.0)	
35–49	2440	(27.6)	185	(7.6)	2255	(92.4)		3103	(31.8)	203	(6.5)	2900	(93.5)	
50≤	4636	(52.4)	515	(11.1)	4121	(88.9)		4670	(47.8)	369	(7.9)	4301	(92.1)	
Education Level							<0.0001							0.0024
Middle school or lower	2125	(24.0)	277	(13.0)	1848	(87.0)		2949	(30.2)	265	(9.0)	2684	(91.0)	
High School	3105	(35.1)	259	(8.3)	2846	(91.7)		3208	(32.8)	217	(6.8)	2991	(93.2)	
University degree or higher	3612	(40.9)	282	(7.8)	3330	(92.2)		3611	(37.0)	250	(6.9)	3361	(93.1)	
Income Level							<0.0001							<0.0001
Low	1409	(15.9)	202	(14.3)	1207	(85.7)		1604	(16.4)	159	(9.9)	1445	(90.1)	
Middle Low	2108	(23.8)	210	(10.0)	1898	(90.0)		2415	(24.7)	188	(7.8)	2227	(92.2)	
Middle High	2513	(28.4)	187	(7.4)	2326	(92.6)		2806	(28.7)	211	(7.5)	2595	(92.5)	
High	2812	(31.8)	219	(7.8)	2593	(92.2)		2943	(30.1)	174	(5.9)	2769	(94.1)	
Marital Status							0.9107							0.1904
Married	6457	(73.0)	596	(9.2)	5861	(90.8)		6644	(68.0)	482	(7.3)	6162	(92.7)	
Unmarried	2385	(27.0)	222	(9.3)	2163	(90.7)		3124	(32.0)	250	(8.0)	2874	(92.0)	
Job Classification							<0.0001							<0.0001
White	2613	(29.6)	195	(7.5)	2418	(92.5)		2351	(24.1)	134	(5.7)	2217	(94.3)	
Pink	928	(10.5)	71	(7.7)	857	(92.3)		1574	(16.1)	90	(5.7)	1484	(94.3)	
Blue	2968	(33.6)	277	(9.3)	2691	(90.7)		1448	(14.8)	92	(6.4)	1356	(93.6)	
Unemployed	2333	(26.4)	275	(11.8)	2058	(88.2)		4395	(45.0)	416	(9.5)	3979	(90.5)	
Region							0.9351							0.3958
Urban	4128	(46.7)	383	(9.3)	3745	(90.7)		4671	(47.8)	339	(7.3)	4332	(92.7)	
Rural	4714	(53.3)	435	(9.2)	4279	(90.8)		5097	(52.2)	393	(7.7)	4704	(92.3)	
Smoking							0.0271							0.0020
Never	1945	(22.0)	155	(8.0)	1790	(92.0)		8572	(87.8)	616	(7.2)	7956	(92.8)	
Ever	6897	(78.0)	663	(9.6)	6234	(90.4)		1196	(12.2)	116	(9.7)	1080	(90.3)	
Drinking							<0.0001							0.0361
Never	1158	(13.1)	144	(12.4)	1014	(87.6)		2179	(22.3)	186	(8.5)	1993	(91.5)	
Ever	7684	(86.9)	674	(8.8)	7010	(91.2)		7589	(77.7)	546	(7.2)	7043	(92.8)	
Aerobic Exercise							0.0082							0.0152
No	4605	(52.1)	462	(10.0)	4143	(90.0)		5534	(56.7)	446	(8.1)	5088	(91.9)	
Yes	4237	(47.9)	356	(8.4)	3881	(91.6)		4234	(43.3)	286	(6.8)	3948	(93.2)	
Subjective Health Condition							<0.0001							<0.0001
Good	2875	(32.5)	185	(6.4)	2690	(93.6)		2584	(26.5)	131	(5.1)	2453	(94.9)	
Normal	4546	(51.4)	433	(9.5)	4113	(90.5)		5176	(53.0)	388	(7.5)	4788	(92.5)	
Bad	1421	(16.1)	200	(14.1)	1221	(85.9)		2008	(20.6)	213	(10.6)	1795	(89.4)	
Number of Chronic Diseases							<0.0001							0.0047
0	5661	(64.0)	440	(7.8)	5221	(92.2)		6884	(70.5)	483	(7.0)	6401	(93.0)	
1	1762	(19.9)	219	(12.4)	1543	(87.6)		1563	(16.0)	130	(8.3)	1433	(91.7)	
≥2	1419	(16.0)	159	(11.2)	1260	(88.8)		1321	(13.5)	119	(9.0)	1202	(91.0)	

^a^ As defined by the Korean Society for the Study of Obesity (KSSO), the cut-off value for waist circumference (WC) is ≥ 90 cm for women and ≥ 85 cm for men; ^b^ As set by the World Health Organization (WHO), a body mass index (BMI) higher than 25 kg/m^2^ indicates general obesity in the Asian population.

**Table 2:** Association of BMI and WC with elevated high-sensitivity C-reactive protein (hs-CRP) levels.

Variables	Elevated hs-CRP Levels ≥3 mg/L			
	Male		Female	
	Adjusted OR	95% CI	Adjusted OR	95% CI
**Obesity**				
Normal	1.00	-	1.00	-
High WC ^a^	1.19	(0.86–1.64)	1.77	(1.24–2.54)
High BMI ^b^	0.99	(0.77–1.29)	2.08	(1.58–2.74)
High BMI & WC ^c^	1.57	(1.33–1.85)	3.70	(3.09–4.43)
**Age**				
20–34	1.00	-	1.00	-
35–49	1.21	(0.92–1.60)	0.73	(0.57–0.92)
50≤	1.50	(1.13–2.00)	0.73	(0.55–0.96)
**Education Level**				
Middle school or lower	1.15	(0.90–1.45)	0.91	(0.69–1.19)
High School	0.94	(0.77–1.15)	0.87	(0.70–1.07)
University degree or higher	1.00	-	1.00	-
**Income Level**				
Low	1.37	(1.07–1.75)	1.18	(0.90–1.54)
Middle Low	1.13	(0.91–1.40)	1.04	(0.83–1.31)
Middle High	0.93	(0.75–1.14)	1.13	(0.92–1.41)
High	1.00	-	1.00	-
**Marital Status**				
Married	1.00	-	1.00	-
Unmarried	1.23	(1.00–1.50)	1.01	(0.84–1.21)
**Job Classification**				
White	1.00	-	1.00	-
Pink	1.12	(0.88–1.43)	1.46	(1.16–1.84)
Blue	1.01	(0.80–1.27)	0.95	(0.69–1.30)
Unemployed	0.94	(0.70–1.27)	0.93	(0.69–1.26)
**Region**				
Urban	1.08	(0.93–1.25)	0.99	(0.85–1.16)
Rural	1.00	-	1.00	-
**Smoking**				
Never	1.00	-	1.00	-
Ever	1.06	(0.88–1.29)	1.21	(0.97–1.51)
**Drinking**				
Never	1.00	-	1.00	-
Ever	0.87	(0.71–1.06)	0.96	(0.80–1.15)
**Aerobic Exercise**				
No	1.02	(0.88–1.19)	1.12	(0.95–1.31)
Yes	1.00	-	1.00	-
**Subjective Health Condition**				
Good	1.00	-	1.00	-
Normal	1.42	(1.18–1.71)	1.40	(1.14–1.73)
Bad	1.86	(1.48–2.33)	1.73	(1.36–2.20)
**Number of Chronic Diseases**				
0	1.00	-	1.00	-
1	1.22	(1.01–1.48)	0.90	(0.71–1.14)
≥2	0.91	(0.73–1.14)	0.74	(0.57–0.96)

^a^ As defined by the Korean Society for the Study of Obesity (KSSO), the cut-off value for waist circumference is ≥ 90 cm for women and ≥ 85 cm for men; ^b^ As set by the World Health Organization (WHO), a BMI higher than 25 kg/m^2^ indicates general obesity in the Asian population; ^c^ Additive analysis results. In men, relative excess risk due to interaction = 0.39; 95% CI (−0.09–0.86), Synergy Index = 3.16 (95% CI 2.69–3.64), Attributable Proportion = 0.25; 95% CI (−0.23–0.72). In women, relative excess risk due to interaction  =  0.85; 95% CI (−0.06–1.75), Synergy Index = 1.46 (95% CI 0.55–2.36), Attributable Proportion = 0.23; 95% CI (−0.68–1.13).
